# Asymmetric Palladium-Catalysed Intramolecular Wacker-Type Cyclisations of Unsaturated Alcohols and Amino Alcohols

**DOI:** 10.3390/molecules18066173

**Published:** 2013-05-24

**Authors:** Jana Doháňošová, Tibor Gracza

**Affiliations:** Department of Organic Chemistry, Slovak University of Technology, Radlinského 9, SK-812 37 Bratislava, Slovakia; E-Mail: tibor.gracza@stuba.sk

**Keywords:** palladium, asymmetric catalysis, Wacker-type reaction, heterocyclisation, oxycarbonylation, lactonisation, chiral ligands

## Abstract

The palladium (II)-catalysed reactions of alkenols and aminoalkenols such as oxycarbonylations or bicyclisations are powerful methods for the construction of oxygen and nitrogen-containing heterocyclic compounds. This review highlights recent progress in the development of the asymmetric palladium(II)-catalysed Wacker-type cyclisations of unsaturated polyols and aminoalcohols. The scope, limitations, and applications of these reactions are presented.

## 1. Introduction

**Scheme 1:**
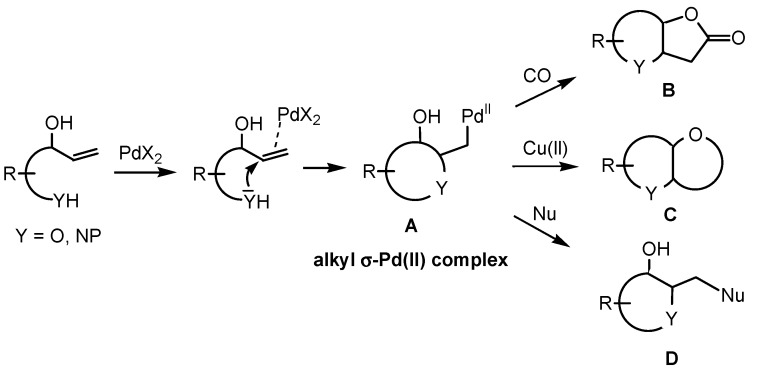
Palladium-catalysed intramolecular Wacker-type cyclisations of unsaturated alcohols and amino alcohols.

Palladium-catalysed functionalisations of alkenes have become a powerful tool in organic synthesis [1,2,3,4]. Today, there are numerous applications of these transformations in the preparation of a large array of the useful products. Among them, an intramolecular oxidative cyclisation, referred to as the Wacker-type cyclisation, is one of the most versatile methods for the preparation of heterocycles [5,6,7]. Particularly, palladium(II)-catalysed reactions of unsaturated alcohols and amino alcohols such as oxy-/aminocarbonylations [8,9,10,11,12,13,14,15], bicyclisations [16,17,18] and domino cyclisation-cross couplings [19,20,21,22,23] serve as a potent stereoselective methods for the construction of oxa-/azaheterocyclic structures found in many natural or biologically relevant compounds [19,20,21,24,25]. The key intermediate of these domino transformations is an alkyl-σ-Pd(II) complex **A**, which is trapped by carbon monoxide and/or by cyclisation with a second hydroxyl function specifically placed in the substrate to give bicyclic products **B**, **C** (Scheme 1). Trapping of the intermediate **A** by other nucleophilic species provides oxa/azaheterocyclic compounds **D** linked with various substituents. Although a number of reviews on palladium-catalysed oxidative cyclisation and issues of stereochemical control already exist [3,4,5,6,7,22,23,24,26,27,28,29,30,31,32,33,34], no general summary on the asymmetric versions of the Pd(II)-catalysed Wacker-type cyclisation of unsaturated alcohols and aminoalcohols has been published. Considering the great potential for application of these methods to the synthesis of biologically active targets and for the extension to the synthesis of optically pure saturated heterocycles, we report here a summary of the main achievements in this area.

## 2. Asymmetric Wacker-Type Oxidative Heterocyclisations

**Scheme 2:**
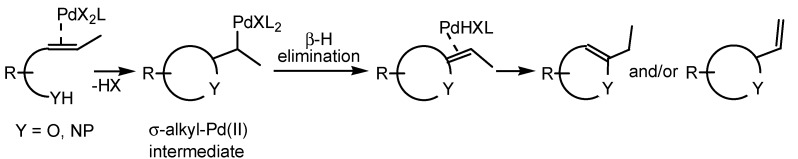
Pd(II)-catalysed Wacker-type oxidative heterocyclisations.

The intramolecular Wacker-type cyclisation using oxygen or nitrogen nucleophiles is one of the most important processes for the preparation of *O*- and *N*-heterocycles [4,5,6]. The palladium(II) coordinates to the alkene C−C double bond and activates it towards nucleophilic attack. Subsequent β-hydride elimination leads to the cyclised product in its thermodynamically stable form (Scheme 2).

### 2.1. Oxidative Wacker Cyclisation of o-Allylphenols

**Scheme 3:**
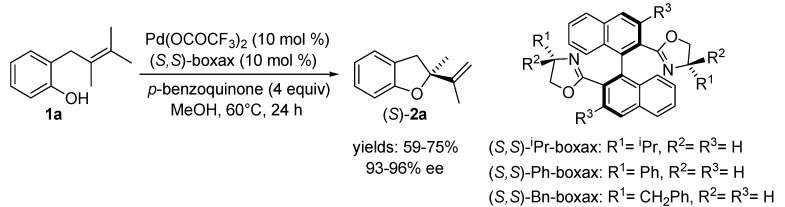
AsymmetricPd(II)-catalysed Wacker-type cyclisation of allylphenol **1a** using (*S*,*S*)-boxax ligands.

Compared to the impressive development of asymmetric reactions with chiral palladium(0) catalysts, asymmetric oxidative reactions with palladium(II) species have received only scant attention. The first attempts to accomplish an asymmetric version of Wacker-type cyclisation were described by Hosokawa and Murahashi [35,36]. However, the truly effective ligands for this transformation were developed by Uozomi and Hayashi [37,38,39]. 2-(2,3-Dimethylbut-2-enyl)phenol (**1a**) was cyclised to the corresponding dihydrobenzofuran (*S*)-**2a** using (*S,S*)-boxax {(*S,S*)-2,2′-bis[4-(alkyl)oxazolyl]-1,1′-binaphthyl} ligands in the presence of *p*-benzoquinone in methanol (Scheme 3). The best selectivity and efficiency gave (*S,S*)-^i^Pr-boxax, the cyclised product was formed with 96% ee in 75% yield. It is noteworthy that the diastereomeric isomer (*R,S*)-^i^Pr-boxax (R^1^ = H, R^2^ = ^i^Pr) was much less active and less enantioselective (18% ee, 3% yield).

Another significant feature of this transformation is the strong dependence of catalytic activity on the nature of the anionic ligands attached to the palladium. The reaction of **1a** was much faster with the palladium catalyst generated from palladium bis(trifluoroacetate) than that from palladium diacetate or dichlorobis(acetonitrile)palladium. Furthermore, this reaction was not catalysed by chloride complex PdCl_2_{(*S,S*)-^i^Pr-boxax} at all. Thus, it was expected that a cationic palladium/boxax complex was generated as the active species by dissociation of palladium bis(trifluoroacetate) to the relatively stable trifluoroacetate anion in polar solvent. Indeed, a cationic palladium(II)/boxax species generated by addition of 2 equiv. of (*S,S*)-^i^Pr-boxax to Pd(CH_3_CN)_4_(BF_4_)_2_ was found to be catalytically much more active than the Pd(OCOCF_3_)_2_{(*S,S*)-^i^Pr-boxax} complex. The reaction of **1a** in the presence of mentioned cationic species was complete in 50 min, giving 91% yield of (*S*)-**2a** with 97% ee. Generation of cationic species by abstraction of chloride from PdCl_2_{(*S,S*)-^i^Pr-boxax} through treatment with 2 equiv. of a silver(I) salt (AgBF_4_, AgPF_6_ or AgSbF_6_) was also successful (full conversions of **1a** were achieved in 1 hour to give the product in 86%–91% yield with 95%–98% ee).

**Scheme 4:**
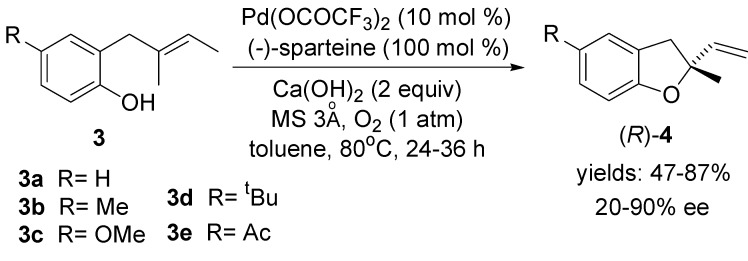
Pd(II)-catalysed asymmetric aerobic oxidative cyclisation of allylphenol **3**.

The Stoltz laboratory has developed an enantioselective Pd(II)-catalysed oxidative phenol cyclisation in nonpolar organic solvents with molecular oxygen using (−)-sparteine as the chiral ligand [40,41] (Scheme 4). 

**Figure 1:**
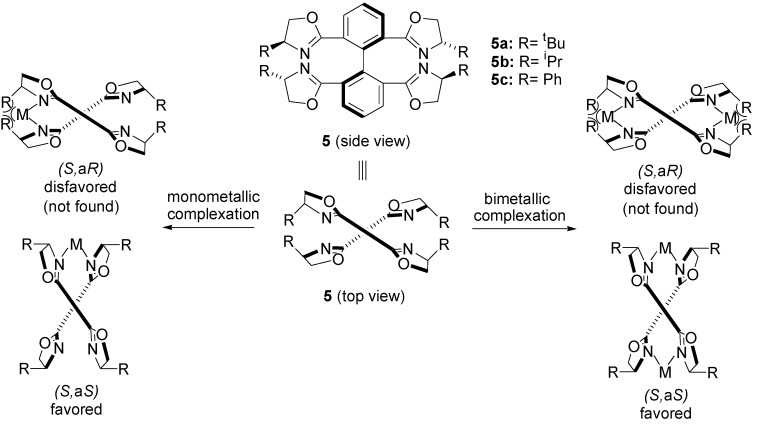
Model figures of diastereomeric monometallic and bimetallic complexes with tetraoxazoline ligands.

From recent works on enantioselective Wacker-type cyclisation of the *o*-allylphenols [42,43,44,45,46], the one worthy of emphasis, published by Zhang [44,45], reports a new family of tetraoxazoline ligands **5** for the construction of chelation-induced axially chiral catalytic systems (Figure 1). The axially achiral tetraoxazoline ligands **5**, in which four identical chiral oxazoline groups are induced into the four *ortho* positions of a biphenyl axis, may produce only one of two possible diastereomeric metal complexes during the coordinating process. As it can be seen in Figure 1, the metal complexes (*S*,a*S*) are sterically more favorable compared with their diastereomers (*S*,a*R*). Hence, it is expected that only one diastereomeric metal complex with (*S*)-axial configuration is formed during the chelation-induced process.

**Scheme 5:**
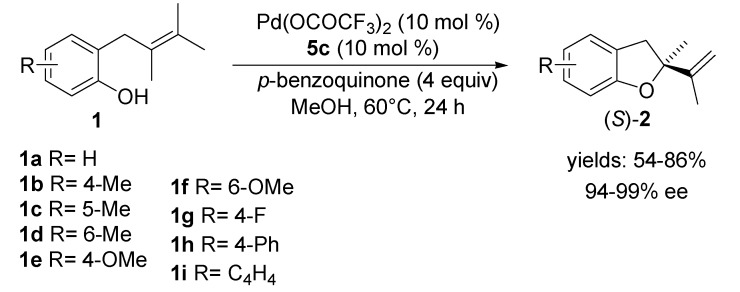
Asymmetric intramolecular Wacker-type cyclisation of **1** using tetraoxazoline **5c**-Pd(OCOCF_3_)_2_ catalyst.

The generality of the chelation-induced axially chiral Pd-catalyst, **5c**-Pd(OCOCF_3_)_2_, has been successfully demonstrated through the Wacker-type cyclisation of a series of *o*-allylphenols **1a**–**h** and *o*-allylnaphtol **1i**. As shown in Scheme 5 a wide array of chiral 2,3-dihydrobenzofurans **2a**–**h** and dihydronaphto[1,2-*b*]furan **2i** was obtained with excellent enantioselectivities (up to 99% ee), regardless of the steric or electronic properties of the aromatic moiety on the substrate **2**.

**Scheme 6:**
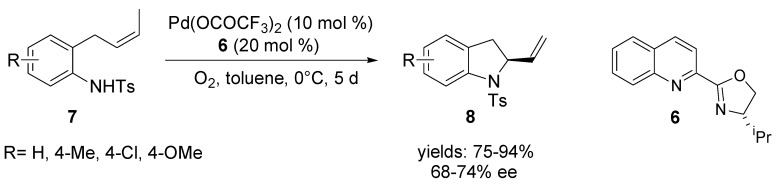
Aza-Wacker-type cyclisation reaction of *o*-allylanilines **7**.

The first Pd-catalysed asymmetric aza-Wacker-type cyclisation of the olefinic tosylamides was published by the same group in 2010 [46]. By using a chiral quinolineoxazoline ligand **6** in the presence of Pd(II)-trifluoroacetate and oxygen at 0 °C, *o*-allylanilines **7** were cyclised to enantiomerically enriched dihydroindoles **8** in good yields with up to 74% ee (Scheme 6).

### 2.2. Oxidative Wacker Cyclisation of Alkeneols

**Scheme 7:**
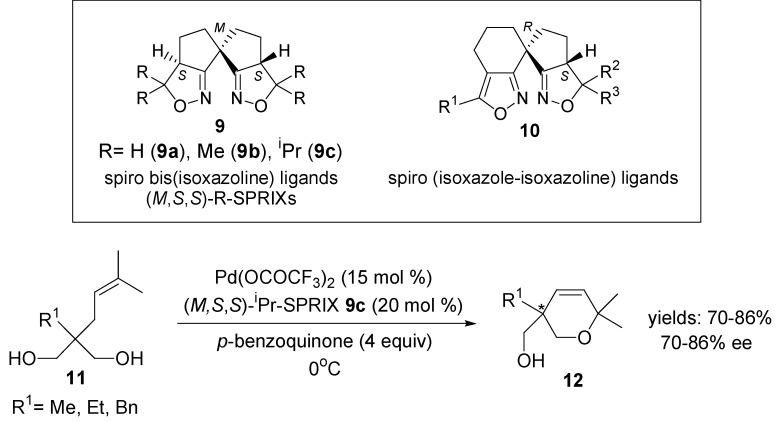

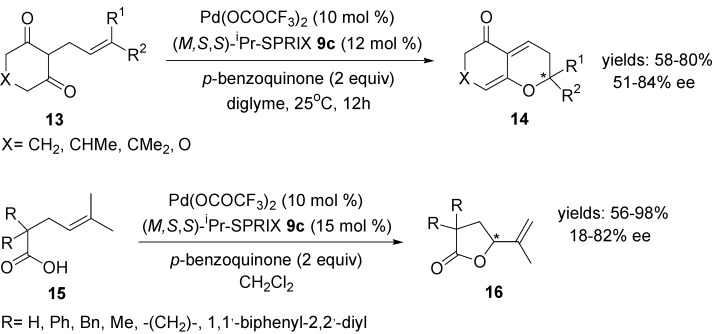
Pd^II^-SPRIX-catalysed cyclisation reaction of alkenyl alcohols **11**, 2-alkenyl-1,3-diketones **13** and 4-alkenoic acids **15**.

The papers of Sasai and co-workers dealing with the development and design of new chiral ligands constitute an outstanding contribution in the field of asymmetric oxidative Wacker-type cyclisation [47,48,49,50,51,52]. Novel spirobis(isoxazoline) **9** and spiro(isoxazole-isoxazoline) ligands **10** were successfully applied in the asymmetric cyclisation of geranylphenols [42], alkenyl alcohols [48,49,50], 2-alkenyl-1,3-diketones [43], 4-alkenoic acids [51] and alkene amides [52]. These transformations effect stereo-selective construction of useful heterocycles (Scheme 7). Dihydropyrans **12** were obtained from alkenyl alcohols **11** with good enantioselectivity (up to 86% ee). Enantioselective 6-*endo-trig* Wacker-type cyclisation of 2-alkenyl-1,3-diketones **13** promoted by Pd-(*M*,*S*,*S*)-^i^Pr-SPRIX catalyst provided optically active chromene derivatives **14**. Recently, the same group published the enantioselective cyclisation of 4-alkenoic acids **15** [51] in this catalytic system. The reaction proceeded via a π-alkyl Pd-intermediate by an allylic C-H activation to give γ-lactone derivatives **16** with moderate to good selectivity.

## 3. Asymmetric Domino Wacker-Type Cyclisation/Coupling Reactions

### 3.1. Coupling with Alkenes via Heck Vinylation

In 1993, Semmelhack showed that an organo-Pd(II) intermediate, formed by intramolecular oxypalladation of hydroxyalkenes, can be trapped by alkenes in the process of Heck vinylation reaction using stoichiometric amount of Pd(OAc)_2_ [53]. From the screening of reoxidation systems for the catalytic version of this transformation, the use of CuCl (1 equiv.)/O_2_ system turned out to be most effective (Wacker conditions). However, this method is limited to substrates that cannot undergo β-hydride elimination from organo-Pd(II) intermediate.

**Scheme 8:**
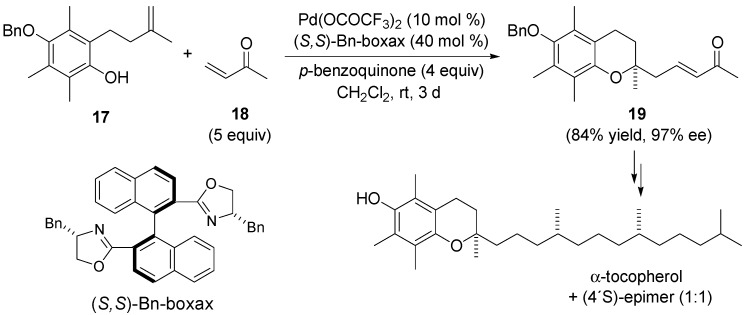
Asymmetric domino Wacker-Heck reaction of alkenyl phenol **17** and methyl vinyl ketone **18** using (*S,S*)-Bn-boxax ligand.

The utility of this domino Wacker-Heck reaction was illustrated by the research group of Tietze in the enantioselective Pd(II)-catalysed total synthesis of vitamin E and stereoselective synthesis of 4-dehydroxydiversonol [19,20,21]. The reaction of alkenyl phenol **17** and methyl vinyl ketone (**18**) dissolved in dichloromethane in the presence of catalytic amount of Pd(OCOCF_3_)_2_, the chiral ligand (*S,S*)-Bn-boxax and *p*-benzoquinone as a reoxidant afforded chromane **19** with 97% ee in 84% yield (Scheme 8). The non-asymmetric synthesis of 2,3-dihydrobenzo[1,4]dioxins, -oxazins, 1,4-dioxanes and perhydro-1,4-oxazines using analogous strategy was also described [54,55].

**Scheme 9:**
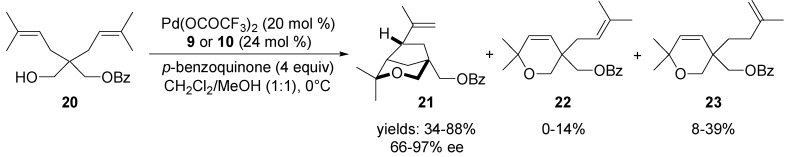
Asymmetric intramolecular domino Wacker-Heck reaction of dialkenyl alcohol **20** using spiro bis(isoxazoline) and spiro (isoxazole-isoxazoline) ligands.

The first enantioselective domino intramolecular Wacker-intramolecular Heck reaction, in which a dialkenyl alcohol was converted into bicyclic ether, was described by Sasai and co-workers [48,49]. In the presence of spirobis(isoxazoline) **9** or spiro(isoxazole-isoxazoline) **10** ligands, the substrate **20** was converted to the domino product **21**, along with dihydropyranes **22** and **23**, products of β-hydride elimination (Scheme 9). Interestingly, previously used catalysts, such as Pd(OCOCF_3_)_2_-bis(oxazolinyl) propane, Pd(OCOCF_3_)_2_-(*S,S*)-^i^Pr-boxax or Pd(OCOCF_3_)_2_-(−)-sparteine did not promote this reaction. This fact was reasoned by a stronger coordinating ability of all these ligands compared with spiro- bis(isooxazolines) **9** and thus, by suppressed Lewis acidity of the catalysts. On the other hand, the chiral spirobis(isoxazole) ligands, with even weaker coordinating ability than SPRIX, were also ineffective in this domino process. Therefore, the spiro(isoxazole-isoxazoline) ligands **10** were designed as a combination of two different coordinating units.

**Scheme 10:**
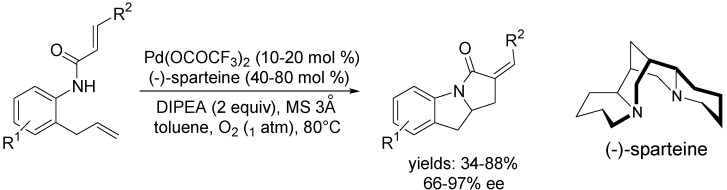
Asymmetric domino Wacker-Heck bicyclisation of 2-allylanilide substrates using (−)-sparteine.

Yang and co-workers [56] reported the enantioselective Pd(II)-catalysed domino Wacker-Heck bicyclisation using nitrogen atom-based nucleophiles and molecular oxygen as the sole reoxidant. Using the chiral Pd(II)-(−)-sparteine complex, 2-allylanilide substrates were converted to corresponding indoline domino products (Scheme 10).

**Scheme 11:**
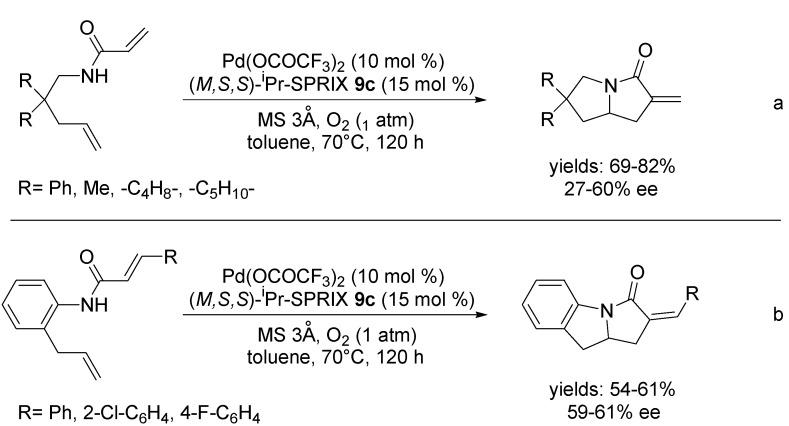
Asymmetric domino Wacker-Heck reaction in the synthesis of pyrrolizines/ pyrroloindoles using (*M,S,S*)-^i^Pr-SPRIX ligand **9c**.

Afterwards, Sasai *et al*. successfully utilised the Pd(II)-SPRIX catalyst in the same reaction for the construction of pyrrolizines/pyrroloindoles [52]. Under the optimised conditions, which is 10 mol % of Pd(OCOCF_3_)_2_, 15 mol % of (*M,S,S*)-^i^Pr-SPRIX **9c** and 3Å MS in toluene at 70 °C under O_2_ atmosphere for 120 h, substrates with *gem*-dialkyl groups, cyclopentyl- or cyclohexyl-substituted substrates (Scheme 11, Equation *a*), and *N*-(2-allylphenyl) cinnamamide, (E)-*N*-(2-allylphenyl)-3-(2-chlorophenyl)-acrylamide and (E)-*N*-(2-allylphenyl)-3-(4-fluorophenyl)acrylamide substrates (Scheme 11, Equation *b*) were tested.

### 3.2. Coupling with Aryl Halides

Wolfe and co-workers developed a general strategy for synthesis of saturated heterocycles via palladium-catalysed carboetherification and carboamination reactions between aryl or alkenyl halides and alkenes bearing pendant heteroatoms [22,23,57]. These transformations effect the stereoselective construction of synthetically interesting heterocycles, such as tetrahydrofurans, pyrrolidines, imidazolidin-2-ones, isoxazolidines, oxazolidines, pyrazolidines, piperazines, morpholines and diazepines.

**Scheme 12:**
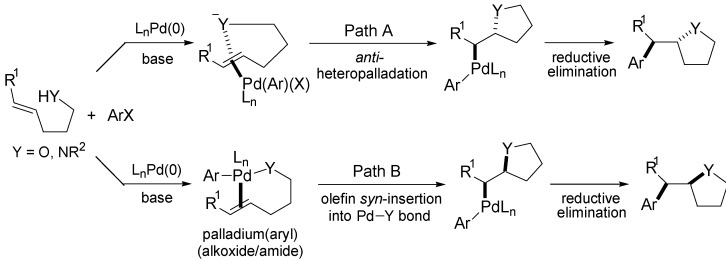
Mechanism of palladium-catalysed carboetherification and carboamination reactions.

One of the possible mechanisms involves the coordination of the alkene to the Pd(Ar)(X) species [generated upon oxidative addition of the aryl halide to Pd(0)], which activates the double bond toward nucleophilic attack (Scheme 12, Path A). The alkyl-σ-Pd(II) complex formed in the process of *anti*-heteropalladation is subsequently converted to the product through the well-known carbon–carbon bond forming reductive elimination. *anti*-Heteropalladation reactions are well-established with relatively electrophilic PdX_2_ complexes, however, these processes are not as common with less-electrophilic Pd(Ar)(X) intermediates [58,59]. Another plausible mechanism could proceed through oxidative addition of the aryl halide to Pd(0) followed by substrate deprotonation and substitution to provide alkene-bound palladium(aryl)(alkoxide/amide) complexes (Scheme 12, Path B). The alkyl-σ-Pd(II) complex is then formed in the process of *syn*-1,2-migratory insertion into the Pd–O/N bond and converted to the product by reductive elimination [22,23,57].

**Scheme 13:**
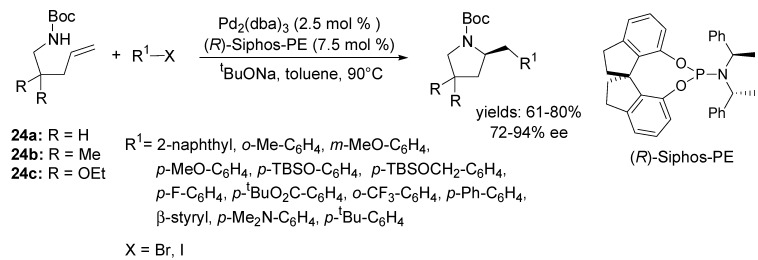
Asymmetric palladium-catalysed carboamination reaction for the synthesis of enantiomerically enriched pyrrolidines using (*R*)-Siphos-PE ligand.

The first asymmetric variant of this methodology was developed by Wolfe and Mai for the synthesis of enantioenriched pyrrolidines [60]. The substrates **24a**–**c** were coupled with several different aryl or alkenyl bromides and iodides using (*R*)-Siphos-PE as ligand to give the desired products in moderate to good yields with 72%–94% ee (Scheme 13). Interestingly, little or no stereocontrol was observed with chiral bidentate ligands.

**Scheme 14:**
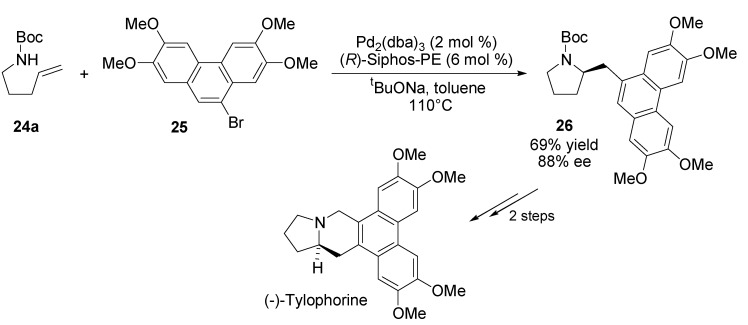
Synthesis of (–)-tylophorine.

The asymmetric carboamination method was applied towards a concise enantioselective synthesis of (–)-tylophorine [60]. Aryl bromide **25** was coupled with *N*-boc-pent-4-enylamine (**24a**) using the Pd/(*R*)-Siphos-PE catalyst (Scheme 14). The desired pyrrolidine **26** was formed in 69% yield and 88% ee, and converted to (–)-tylophorine in two steps and nearly quantitative yield.

**Scheme 15:**
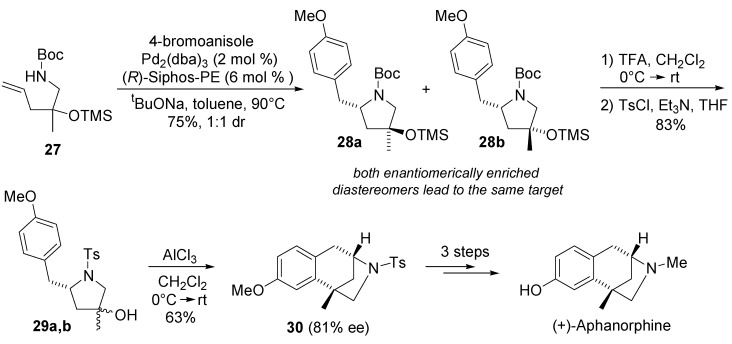
Synthesis of (+)-aphanorphine.

The utility of asymmetric carboamination method was also illustrated in an enantioconvergent synthesis of the benzomorphan alkaloid (+)-aphanorphine [61]. Racemic γ-aminoalkene derivative **27** and 4-bromoanisole were transformed into a 1:1 mixture of enantiomerically enriched diastereomers **28** (Scheme 15). The treatment of this mixture with trifluoroacetic acid led to cleavage of both the *N*-Boc and *O*-TMS groups, and tosylation of resulting pyrrolidine derivative provided **29a**,**b** in 83% yield (1:1 dr) over two steps. The enantioconvergent intramolecular Friedel-Crafts alkylation of this diastereomeric mixture provided **30** in 63% yield with 81% ee.

**Scheme 16:**
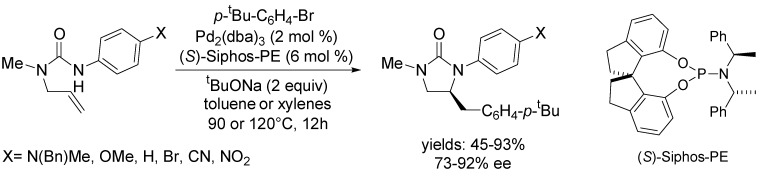
Asymmetric carboamination reaction of *N*-allyl urea derivatives with 1-bromo-4-*tert*-butylbenzene using (*S*)-Siphos-PE ligand.

Most recently, Wolfe and Hopkins described the first asymmetric alkene carboamination reaction between *N*-allyl urea derivatives and aryl halides [62]. The authors initially examined the effect of nitrogen nucleophilicity on asymmetric induction using (*S*)-Siphos-PE ligand (Scheme 16). The level of asymmetric induction increased with increasing electron-withdrawing ability of the *p*-substituent on *N*-aryl moiety, however, the chemical yield decreased due to the diminished reactivity of these substrates. The enhancement of the reaction temperature (120 °C in xylenes) solved the problem with reactivity and the desired products were generated in 81%–87% yield with 86%–92% ee.

**Scheme 17:**
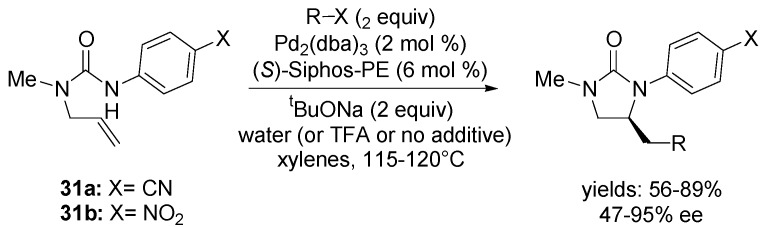
Asymmetric carboamination reaction of substrates **31a**,**b** with different aryl halides using (*S*)-Siphos-PE ligand.

Afterwards, the substrates **31a**,**b** were coupled with a range of different aryl halide derivatives (Scheme 17). It was found that addition of 2 equiv of water (or 40 mol % of TFA) to the reaction mixtures in some cases significantly improved the enantioselectivities. Efforts to employ alkenyl halides as coupling partners were mostly unsuccessful.

## 4. Asymmetric Domino Wacker-Type Cyclisation/Carbonylation Reactions

Palladium(II)-catalysed cylisations of unsaturated alcohols, amines and other suitable substrates accompanied by the insertion of carbon monoxide provide a simple and straightforward access to one-carbon homologated esters, lactones and amides [1,2,6,29,30,31,32,33,34]. These domino processes have proven particularly useful for stereoselective construction of a range of oxygen and nitrogen-containing heterocyclic compounds [7,8,9,10,11,12,13,14,15]. Such a transformation of enantiomerically pure substrates has found numerous applications as the key step in the total syntheses of natural compounds [17,18,25,26,27,28,29].

### 4.1. Intramolecular Alkoxylation/Methoxycarbonylation

**Scheme 18:**
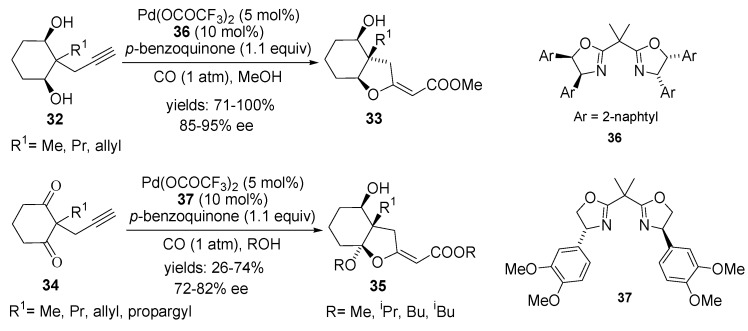
Asymmetric cyclisation-carbonylation of *meso*-2-alkyl-2-propargyl-1,3-cyclohexane-diols **32** and -1,3-diones **34** using Pd(II)-[box] catalysts.

The first asymmetric version of the domino Pd-catalysed cyclisation-carbonylation reaction reported Kato, Akita *et al*. accomplishing desymmetrisation of cyclic *meso*-2-methyl-2-propargyl-cyclohexane-1,3-diols **32** [63] and -1,3-diones **34** [64] using palladium(II)-complex, bearing chiral bis(oxazoline) ligands (Scheme 18). Based on a ligand screening, by using Pd(OCOCF_3_)_2_-{2,2′-isopropylidenebis[4*S*,5*R*)-4,5-di(2-naphtyl)-2-oxazoline] (**36)**} catalyst in the presence of *p*-benzoquinone in methanol at −45 °C to −50 °C under carbon monoxide atmosphere, the cyclic *meso*-2-alkyl-2-propargyl-1,3-cyclohexane-diols **32** were carbonylated to bicyclic-alkoxyacrylates **33** in good yields with high enantioselectivities (85%–95% ee) [65]. The best results for desymmetrisation of cyclohexane-diones **34** were achieved with Pd(OCOCF_3_)_2_-{2,2′-isopropylidenebis[(4*R*)-4-(3,4-dimethoxyphenyl)-2-oxazoline] (**37**)} affording enantiomerically enriched bicyclic products **35** in 28%–74% yields with 72%–82% ee [66]. 

**Scheme 19:**
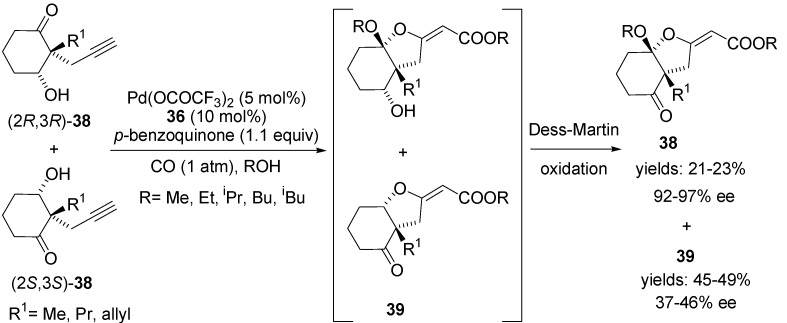
Parallel kinetic resolution of propargyl ketols **38** using Pd(II)-[box] catalyst.

Further expansion of this oxidative cyclisation-carbonylation domino process into asymmetric transformations has been demonstrated in the parallel kinetic resolution of propargyl ketols **38** (Scheme 19). The 2*S*,3*S* enantiomer of (±)-**38** was preferentially converted to **39** (45%–49% yields, 37%–46% ee), and the 2*R*,3*R* enantiomer of (±)-**38** was transformed into **40** by subsequent Dess-Martin oxidation (21%–23% yields, 92%–97% ee) [67].

**Scheme 20:**
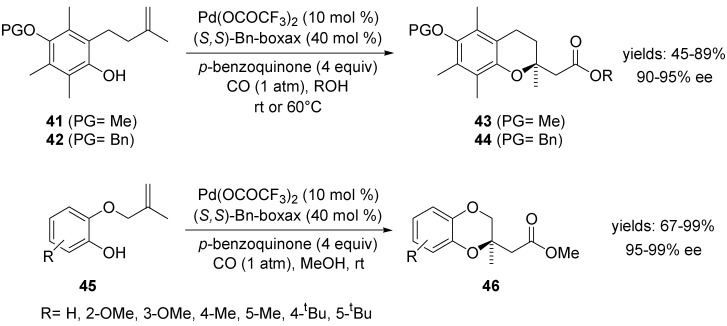
Asymmetric domino reaction of alkenes **41**–**42** and phenyl ethers **45** employing (*S,S*)-Bn-boxax.

In 2007, Tietze and co-workers described an enantioselective palladium-catalysed domino reaction of alkenes **41** and **42** as well as allyl phenyl ethers **45** affording the corresponding chromans **43**, **44** and benzodioxins **46** (Scheme 20) [68]. In some cases, better results were obtained when the reaction was performed in dichloromethane with only 1 equiv of ROH. In other few cases, the use of molybdenium hexacarbonyl as CO-source instead of carbon monoxide at ambient pressure was advantageous.

**Scheme 21:**
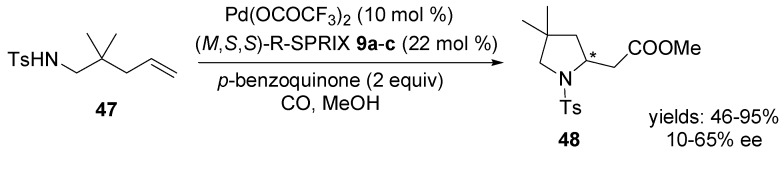
Asymmetric aminocarbonylation of *N*-(2,2-dimethylpent-4-enyl)-*p*-toluenesulfonamide **47** using Pd(II)-[SPRIX] catalysts.

The first enantioselective aminocarbonylation was published by Sasai and co-workers [69]. The reaction of *N*-(2,2-dimethylpent-4-enyl)-*p*-toluenesulfonamide **47** in the presence of Pd(II)-SPRIX catalysts and *p*-benzoquinone in methanol under a carbon monoxide atmosphere afforded the corresponding pyrrolidinyl acetic acid methyl ester **48** in a good yield and moderate enantioselectivity (Scheme 21).

### 4.2. Intramolecular Alkoxylation/Lactonisation

**Scheme 22:**
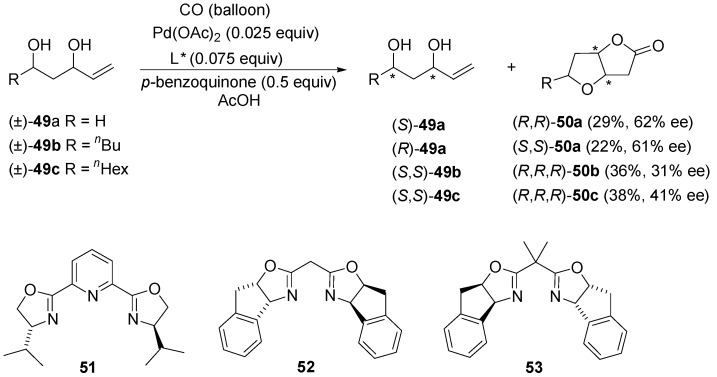
Kinetic resolution of alkene-1,3-diols (±)**-49** in an asymmetric Pd(II)-catalysed oxycarbonylation.

In 2008, Gracza and co-workers [70,71] disclosed an enantioselective variant of another Wacker-type Pd(II)-catalysed cyclisation. The oxycarbonylative annulations of unsaturated polyols and amino alcohols, either diastereoselective or enantioselective, proceeded to bicyclic lactones using chiral palladium(II) complexes. In the initial report [70], a method for the kinetic resolution of alkene-α,γ-1,3-diols **49** via asymmetric oxycarbonylative bicyclisation has been investigated. The conversion was controlled by the amount of *p-*benzoquinone. Besides the chiral ligand, the efficiency of this process depends on the anionic part of the catalyst and the solvent. Based on a ligand screening, the box-type *N*,*N*-bidentate ligands 2,6-bis[(*R*)-4-phenyloxazolin-2-yl]pyridine **51**, {(3a*R*,8a*S*)-bis(8,8a-dihydro-3a*H*-indeno[1,2-*d*]oxazol-2-yl)}methane (**52**) and {(3a*S*,8a*R*)-bis(8,8a-dihydro-3a*H*-indeno[1,2-*d*]-oxazol-2-yl)}isopropane (**53**) have been identified as the most suitable ligands for Pd-catalysed oxidative lactonisation of unsaturated diols (Scheme 22). Sulphur and/or phosphorus-containing ligands have been proven to be incompatible with the oxidative catalytic system. Under optimum conditions, the kinetic resolution of pent-4-ene-diol (±)-**49a** using Pd(II)-[(*R*,*S*)-indabox] (**51**) and Pd(II)-[(*S*,*R*)-indabox] (**53**) provided both enantiomerically enriched lactones (*R*,*R*)-**50a** (29% yield, 62% ee) and (*S*,*S*)-**50a** (22% yield, 61% ee), respectively [71]. Similarly, the *syn*-diols (±)**-49b** and (±)**-49c** afforded the corresponding natural Hagen’s glands *exo*-lactones (*R*,*R,R*)-**50b** and (*R*,*R,R*)-**50c**, with high diastereoselectivity and good yields, however with low enantioselectivities. It should be noted that, contrary to CuCl_2_, the regeneration of the active Pd(II) species with *p-*benzoquinone is carried out in the absence of AcONa. 

**Scheme 23:**
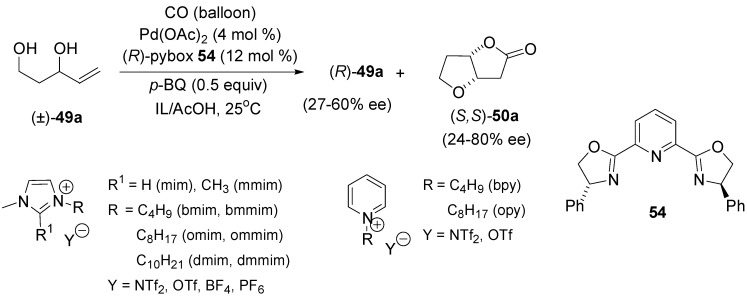
Kinetic resolution of pent-4-ene-1,3-diol (±)**-49a** by Pd(II)-catalysed oxycarbonylation in ionic liquids.

Efforts from Vo-Thanh’s and Gracza’s group improved the efficiency of the process [71]. By application of ionic liquids and/or microwave activation, noticeable propitious enhancements in both reaction rate and enantiomeric excess (up to 80% ee) were observed (Scheme 23).

**Scheme 24:**
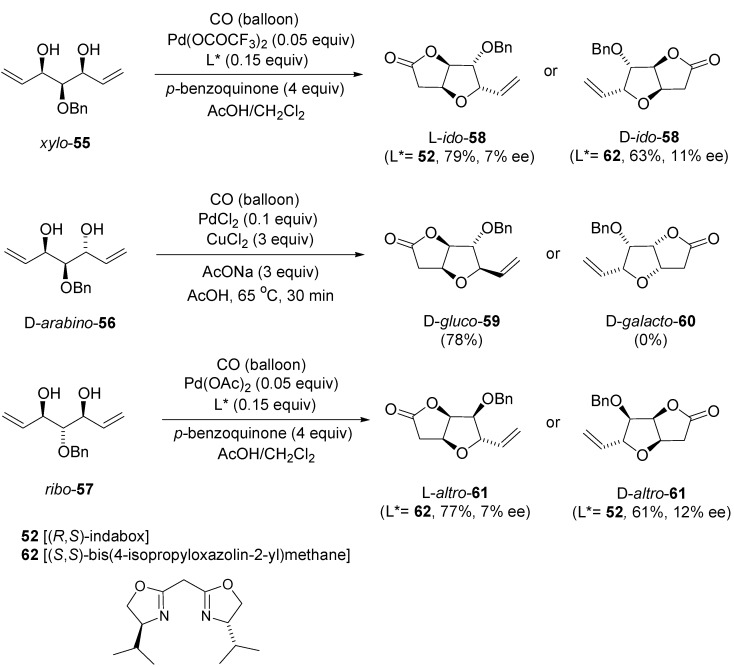
Palladium(II)-catalysed oxycarbonylation of *meso*-diols **55** and **57**, and *pseudo*-C_2_-symmetric enitol **56**.

In a later study of the transformation of symmetric substrates [72], the same authors reported Pd-catalysed oxycarbonylation of the *meso*-diols *xylo*-**55**, *ribo*-**57** and *pseudo*-C_2_-symmetric d-*arabino*-derivative **56**. The Pd(II)-initiated oxycarbonylative bicyclisation of *meso*-diols **55**, **57** in the presence of chiral Pd-catalysts using ligands with opposite asymmetric induction [(*R*,*S*)-indabox **52**, (*S*,*S*)-bis(4-isopropyloxazolin-2-yl)methane (**62**)] afforded bicylic lactones **58** and **61** in good yields and with excellent 2,3-*threo*-diastereoselectivity (Scheme 24). In the reaction of the of the *pseudo*-C_2_-symmetric enitol **56**, the diastereomer d-*gluco***-59** was isolated as a major product (65% yield, resulting from the intramolecular *Si*-attack of nucleophilic hydroxyl group to the Pd(II)-activated double bond) along with its minor diastereomer d-*galacto*-**60** (9%). Such product distribution is most probably due to the *endo*-positions of two attached substituents on fused rings of lactone d-*galacto*-**60**, which in consequence increased the steric hindrance, and therefore its formation is slowed down. In fact, simply raising of temperature to 60 °C led to the complete consumption of enitol **56** in only 30 min to form energetically favorable diastereomer d-*gluco*-**59** as the sole product in 78% yield. 

**Scheme 25:**
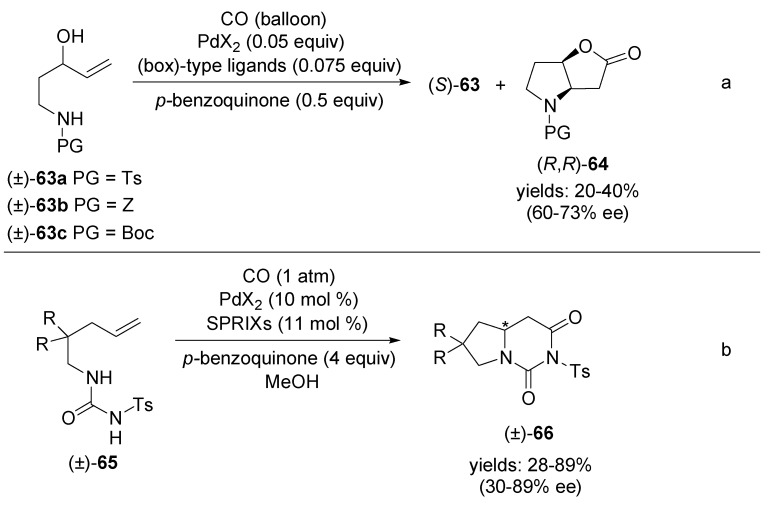
Kinetic resolution of *N*-protected 1-aminopent-4-ene-3-ols (±)**-63** in the Pd(II)-catalysed amidocarbonylation and Pd(II)-catalysed amidocarbonylation of alkenylureas **65**.

Recently, asymmetric Pd(II)-catalysed carbonylative bicyclisation of amino alcohols have been disclosed [73,74]. Gracza and co-workers reported the kinetic resolution of racemic *N*-protected 1-amino-pent-4-ene-3-ols **63** catalysed by *prior to use* prepared chiral palladium(II) complexes [73]. The *N*-protected 2-oxa-6-azabicyclo[3.3.0]octan-3-ones (*R*,*R*)**-64** (derivatives of the natural Geissman-Waiss lactone) were obtained in 20%–40% yields with 60%–73% ee. (Scheme 25a). Sasai and co-workers succeeded in constructing the tetrahydropyrrolo[1,2-*c*]pyrimidine skeleton **66** via Pd(II)-catalysed amidocarbonylation of alkenylureas **65** (Scheme 25b) [74]. By using the chiral spiro bis(isoxazoline) ligands, SPRIXs, the desired products of carbonylative bicyclisation were produced in good yields and with moderate to good enantioselectivities.

## 5. Conclusions

The palladium(II)-catalysed Wacker-type cyclisations of alkenes have evolved into a highly useful methodology in synthetic organic chemistry. The domino and/or multicatalytic processes involving intramolecular oxidative cyclisation reactions, employing oxygen/nitrogen-containing nucleophiles, can provide various heterocyclic compounds. In many cases, the chemo-, regio- and diastereoselective Pd(II)-mediated cyclisations have succeeded in building of complex structures. This overview summarised the asymmetric versions of these palladium(II)-catalysed processes. The main issues include: (1) asymmetric Wacker-type oxidative cyclisations of substrates having both a carbon-carbon double bond and an amino/hydroxylated tether; (2) asymmetric domino Pd-catalysed *N*/*O*-cyclisation/coupling reactions; (3) asymmetric intramolecular alkoxylation/ methoxycarbonylation; (4) asymmetric intramolecular alkoxylation/lactonisation. Although considerable progress has been made in this area, some future trends are easy to predict: new catalytic systems will be developed to make Pd(0)-reoxidation more efficient, and a larger variety of chiral ligands will be available for enantioselective transformations. There are also many opportunities for the development of further domino processes and new modifications of the reaction system, omitting carbon monoxide atmosphere for extended use in medicinal chemistry exploiting automated workflows for liquid-phase parallel synthesis. In addition, there is a great potential for the application of these methods for the synthesis of natural products and biologically active heterocycles. 
